# Pathological and Immunohistochemical Characterization of Follicular Gastritis (Gastric Lymphofollicular Hyperplasia) in 41 Dogs

**DOI:** 10.3390/ani14243605

**Published:** 2024-12-14

**Authors:** Andrada Negoescu, Corina Toma, Claudiu Gal, Constantin Ifteme, Bianca Bofan, Teodoru Soare, Irina Amorim, Raluca Maria Pop, Ştefan Cristian Vesa, Dragoș Hodor, Elvira Gagniuc, Cornel Cătoi, Marian Taulescu

**Affiliations:** 1 Department of Veterinary Pathology, Faculty of Veterinary Medicine, University of Agricultural Sciences and Veterinary Medicine Cluj-Napoca, 400372 Cluj-Napoca, Romania; corina.toma@usamvcluj.ro (C.T.); gal.claudiu@gmail.com (C.G.); iamorim@ipatimup.pt (I.A.); dragos.hodor@usamvcluj.ro (D.H.); cornel.catoi@usamvcluj.ro (C.C.); marian.taulescu@usamvcluj.ro (M.T.); 2 Department of Veterinary Pathology, Synevovet, 81 Pache Protopopescu, 021408 Bucharest, Romania; elvira.gubceac@gmail.com; 3 Endoscopy and Minimal Invasive Surgery Veterinary Center, 077190 Bucharest, Romania; centru@endoscopieveterinara.ro (C.I.); dr.bofan@icloud.com (B.B.); 4 Histovet, 050855 Bucharest, Romania; teodoru.soare@gmail.com; 5 Department of Pathology and Forensic Medicine, University of Agronomic Sciences and Veterinary Medicine of Bucharest, 59 Marasti Blvd., 011464 Bucharest, Romania; 6 Department of Pathology and Molecular Immunology of the Institute of Biomedical Sciences Abel Salazar, University of Porto, 4050-313 Porto, Portugal; 7 Pharmacology, Toxicology and Clinical Pharmacology, Department of Morphofunctional Sciences, “Iuliu Haţieganu” University of Medicine and Pharmacy, Victor Babeș, 400012 Cluj-Napoca, Romania; raluca.pop@umfcluj.ro (R.M.P.); stefan.vesa@umfcluj.ro (Ş.C.V.)

**Keywords:** dogs, *Helicobacter* spp., immunohistochemistry, gastritis, lymphoid hyperplasia

## Abstract

Gastric lymphofollicular hyperplasia (GLFH) is characterized by large lymphoid nodules in the lamina propria. Its etiology and immunohistochemical characteristics are poorly understood. This study analyzed 41 canine GLFH cases, including clinical, endoscopic, histopathological, and immunohistochemical evaluations. Young French Bulldogs (75.06%) were the most affected. Endoscopically, lymphoid nodules were identified in both the antrum and gastric body. Lymphoid follicle diameters were similar in the gastric body (mean 295.587 μm) and antrum (mean 294.641 μm). Associated lesions included glandular atrophy, lymphoplasmacytic inflammation, and fibrosis. Minimal, moderate, and severe colonization with *Helicobacter*-like organisms (HLOs) were observed in 20, 6, and 3 cases, respectively. B-cell lymphocytes positive for Bcl6 and Pax5 were localized centrally in large follicles, surrounded by CD3+ T lymphocytes. Small follicles lacked germinal centers and showed mixed T and B lymphocytes. A positive correlation was found between the follicle diameter and both HLOs colonization (*p* = 0.049) and follicular hyperplasia (*p* < 0.001). A regression analysis indicated that HLOs colonization and hyperplasia accounted for 42.3% of follicle diameter variance (R^2^ = 0.423, *p* < 0.001). Additional studies are required to investigate potential correlations between GLFH and HLOs, as well as to assess the role of this lesion in the progression to neoplasia.

## Simple Summary

This study of the gastric mucosa biopsies of 41 dogs highlighted the endoscopic, histopathological, and immunohistochemical findings of gastric lymphofollicular hyperplasia (GLFH), emphasizing its frequent occurrence in young French Bulldogs (75.06%). GLFH was associated with glandular atrophy, lymphoplasmacytic inflammation, fibrosis, and varying degrees of *Helicobacter*-like organisms (HLOs) colonization. Immunohistochemistry showed positive Bcl6 and Pax5 B lymphocytes within the germinative center of large follicles, surrounded by CD3+ T lymphocytes, consistent with hyperplastic changes. A significant correlation was observed between the follicle diameter and HLO colonization (*p* = 0.049) and follicular hyperplasia severity (*p* < 0.001). These findings contribute to the pathogenesis of GLFH in dogs and underscore the need for further studies on its potential progression to cancer.

## 1. Introduction

Chronic gastritis (CG) represents a progressive inflammatory process of the gastric mucosa, characterized clinically by recurrent episodes of vomiting persisting for more than 2 weeks [1,2]. The definitive diagnosis of CG requires a histological evaluation of gastric biopsies, and the inflammatory subtypes are mainly classified based on the predominant inflammatory cells and etiology [2]. Thus, CG is classified into eosinophilic, lymphoplasmacytic, histiocytic/granulomatous, and lymphoid follicular [3]. Other morphological subtypes of gastritis, such as sclerosing (fibrosing), atrophic, and hypertrophic, are well described in dogs [1,2,3,4].

Follicular gastritis (FG) (gastric lymphofollicular hyperplasia (GLFH), lymphofollicular gastritis, nodular lymphoid hyperplasia (NLH), nodular gastritis) is a chronic inflammation of the lamina propria of gastric mucosa, consisting of multifocal mononuclear inflammatory infiltrates with formation of lymphoid follicles showing a mitotically active germinal center and well-defined lymphocyte mantles in the lamina propria [5], with or without intraepithelial lymphocytes [6]. The term “nodular gastritis”, used to describe the milliary appearance of the gastric mucosa during an endoscopy, is not currently included in the Sydney classification of gastritis in humans and it is not yet widely accepted by pathologists worldwide [7]. Histologically, in order to confirm the diagnosis of GLFH, at least two secondary lymphoid follicles (with germinal center) should be identified in an area of 1 cm^2^ within the gastric mucosa [8].

In human patients, GLFH and antral nodularity are highly correlated with *Helicobacter pylori* (*H. pylori*) infection, and the prevalence of follicular gastritis is slowly reduced with increasing age [9,10]. Moreover, nodular gastritis associated with *H. pylori* infection may evolve to diffuse-type of gastric cancer in young women [11] and gastric lymphoma [12]. 

The clinical significance of GLFH in animals is still controversial. In healthy domestic cats, up to 10% of individuals may show lymphoid follicles in the gastric mucosa. Chronic gastritis with lymphofollicular hyperplasia in cats may be induced by infection with *H. pylori* and *H. felis*, where the organisms appear to provide chronic antigenic stimulation resulting in the formation of gastric lymphoid follicles [13,14,15]. Furthermore, chronic infection with *H. pylori* in cats has many similarities to long-term *H. pylori* infection in humans, including the development of diffuse lymphofollicular and atrophic gastritis with areas of mucosal epithelial dysplasia in the antrum and its progression to cancer [16]. 

In dogs, the cause of GLFH and its progression to gastric cancer are still unknown. Canine GLFH was experimentally induced by infection with *H. felis* [17] and *H. pylori* in laboratory Beagles [18]. The disease is also caused by natural infection with *Helicobacter-*like organisms (HLOs), with the severity of GLFH being directly proportional to the extent of HLOs colonization [19]. Recently, an association between inspiratory dyspnea related to brachycephalic phenotype and CG with follicular hyperplasia was found [20]. Lymphoid follicles were also described in the gastric mucosa of dogs without macroscopical changes; the authors considered that lymphoid follicles are a normal constituent of the canine gastric mucosa [21].

The differential diagnosis of GLFH includes other inflammatory lesions (e.g., eosinophilic and histiocytic/granulomatous gastritis), gastric polyps, and malignant lymphoma. GLFH may resemble both clinically and histologically malignant follicular lymphoma. The lesions can be differentiated based on the polymorphic nature of the infiltrate, the absence of significant cytologic atypia, the presence of reactive follicles within gastric mucosa, and by the use of immunohistochemical or molecular analyses [18,22]. 

In GLFH, the reactive lymphoid follicles are composed of a germinative center represented by B cells surrounded by CD3 positive T cells. Lymphoid B-cell identity and the formation of germinal centers are usually regulated by Paired box protein 5 (Pax-5) and B-cell lymphoma 6 protein (BCL6) [23,24]. In contrast to GLFH, the primary follicular lymphoma of the gastrointestinal tract is composed of lymphoid proliferation in the lamina propria, in which the neoplastic cells are positive for B cells’ immunomarkers including CD20, Bcl2 (oncogene), and Bcl6, and negative for CD3 [25]. 

To the best of our knowledge, the immunohistochemical characterization of naturally occurring GLFH in dogs has not been previously described. In this study, we retrospectively analyzed the pathological and immunohistochemical features of GLFH in 41 dogs diagnosed with nodular gastric lesions during an endoscopy. Furthermore, a correlation between GLFH, associated gastric mucosal changes, and the presence of HLOs was also performed. 

## 2. Materials and Methods

### 2.1. The Animals and Sample Collection

Gastric biopsies of forty-one dogs, sampled during endoscopic procedures, were selected from the archives of the Department of Anatomic Pathology, Faculty of Veterinary Medicine Cluj-Napoca (Romania) and Synevovet Laboratories (Bucharest, Romania). The samples were received and analyzed between January 2018 and September 2023. This study was approved by The Research Ethics Committee University of Agricultural Sciences and Veterinary Medicine Cluj-Napoca (Romania), authorization N° 302/2022. The medical procedures were performed in a clinical context, for routine diagnostic workup, and the owners provided written informed consent that samples will be used for this research. Information regarding epidemiological data (breed, gender, age) and endoscopic features of the gastric mucosa were collected for each dog. All endoscopic examinations were performed by two clinicians (C.I. and B.B.). The selection criteria of tissue samples included the following: (1) all cases were histologically diagnosed with follicular gastritis; (2) all gastric samples were well preserved and collected from the body and antrum zones; and (3) histochemistry and immunohistochemistry were performed on all tissue samples. The gastric biopsies with extensive areas of necrosis and/or artifacts and dogs diagnosed with gastric tumors were excluded from the study.

### 2.2. Endoscopy 

For the endoscopical examination, the patients with a clinical history of chronic vomiting were restricted from eating and drinking water for 10 h before the procedure, and for brachycephalic breeds, fasting was mandatory for 16–20 h.

Premedication was performed with butorphanol (0.2–0.3 mg/kg) in combination with dexmedetomidine (1–5 mcg/kg) or acepromazine (5–30 mcg/kg, im or iv). Propofol (2–5 mg/kg) with or without midazolam (0.1–0.2 mg/kg iv) or ketamine (0.5 mg/kg iv) was used for induction. Maintenance was performed with oxygen and sevoflurane or isoflurane on the endotracheal tube. All patients were intravenously premedicated with maropitant and antisecretory drugs (omeprazole/pantoprazole) prior to the procedure.

The examination of the patients was on the left lateral side, using a flexible Karl Storz endoscope with an external diameter of 7.9 mm, length of 140 cm, and diameter of the working channel of 2.8 mm. Biopsies were taken using reusable biopsy forceps of 2.2 mm.

Samples were taken from the gastric body and pyloric antrum regions with a special interest on areas with visible macroscopic lesions. The presumptive diagnosis of follicular gastritis could be anticipated on account of the endoscopic examination, being described by the presence of numerous nodules of variable sizes.

### 2.3. Histopathology

Tissue samples (gastric mucosa biopsies) were fixed in 10% neutral-buffered formalin and paraffin embedded. Serial consecutive 3 μm sections were stained with hematoxylin and eosin (H&E), Giemsa, and Warthin–Starry (WS). Assessments of lymphoid follicles and the inflammatory mucosal lesions were scored according to criteria established by the WSAVA Gastrointestinal Standardization Group [4]. The presence of spiral bacteria consistent with gastric HLOs was graded on a scale of 0 (absent), 1 (mild, 1–20 organisms/40×), 2 (moderate, 21–100 organisms/40×), and 3 (severe, over 100 organisms/40×) [4,19,20].

### 2.4. Immunohistochemistry

For immunohistochemical analysis, the primary antibodies selected were the following: rabbit monoclonal anti-CD3 antibody, clone 2GV6 (Ventana Medical Systems, Tucson, Arizona), rabbit monoclonal anti-Pax5 antibody, clone SP34 (Cell Marque, Rocklin, CA, USA), and mouse monoclonal anti-Bcl6 antibody, clone124 (Ventana Medical Systems, Tucson, Arizona). The immunohistochemical processing was performed with an automated immunostainer (Ventana Benchmark ULTRA, Ventana Medical Systems, Tucson, AZ, USA), with all the reagents being dispensed automatically. All the procedures were performed according to the producer’s recommendation of each antibody for the usage with the ultraView Universal DAB Detection Kit (Ventana Medical Systems, Tucson, USA). All the primary antibodies were ready to use, and no endogenous biotin blocker was applied. Healthy lymph node tissue and the tonsils of the dogs were used as positive controls.

The sections were independently examined by three pathologists (M.T, A.N, and C.G.) using an Olympus BX41 microscope and the photomicrographs were taken using an Olympus SP350 digital camera and Stream Basic imaging software, version 1.5.1 (Olympus Corporation, Tokyo, Japan). When there was a divergence of opinion, an agreed diagnosis was reached through simultaneous evaluation at a multi-head microscope (Zeiss Axio Scope A1, Carl Zeiss Microscopy GmbH, München, Germany).

### 2.5. Statistical Analysis

The obtained data were analyzed using MedCalc^®^ Statistical Software version 22.021 (MedCalc Software Ltd., Ostend, Belgium; https://www.medcalc.org). Descriptive statistics, including sample sizes, medians, and standard deviations, were calculated for the study variables. The distribution of the data was assessed using the Shapiro–Wilk test. Given that some data did not meet the assumptions of normality, non-parametric analyses were utilized. Specifically, the Mann–Whitney U test was applied to evaluate possible associations between variables such as breed, gender, and HLOs infection status. Spearman’s rank-order correlation was conducted to assess the relationships between the diameter of the follicles, the grade of follicular hyperplasia, the severity of associated gastric inflammation, and the level of HLOs infection. A multiple linear regression analysis was conducted to examine the combined effect of the follicular hyperplasia grade and HLOs infection grade on follicle diameter. A *p*-value < 0.05 was considered statistically significant.

For the comparative analysis of the antrum and body gastric regions of the various parameters studied, Wilcoxon signed-rank tests and Kruskal–Wallis tests were used.

## 3. Results

Out of the total number of cases (n = 41) included in this study, the majority of GLFH lesions were diagnosed in French Bulldogs, accounting for 23 out of 41 cases (56.09%), followed by Pugs (n = 5), English Bulldogs (n = 2), Mixed Breeds (n = 4), Yorkshire Terriers (n = 2), and one case each in Schnauzer, Westie, Labrador, and Golden Retriever.

GLFH lesions were predominantly diagnosed in male dogs, accounting for 31 out of 41 cases (75.60%), while females were less represented (10/41 cases; 24.40%). The median age was 3.52 years, ranging from 4 months to 9 years. Notably, the youngest reported individuals with GLFH were French Bulldogs. Due to the retrospective nature of our study, besides chronic vomiting, no other relevant information regarding the clinical signs exhibited by the animals were obtained.

### 3.1. Endoscopy 

The main endoscopic changes noted in the evaluated cases are presented in the Supplementary File S1. 

**Figure 1:**
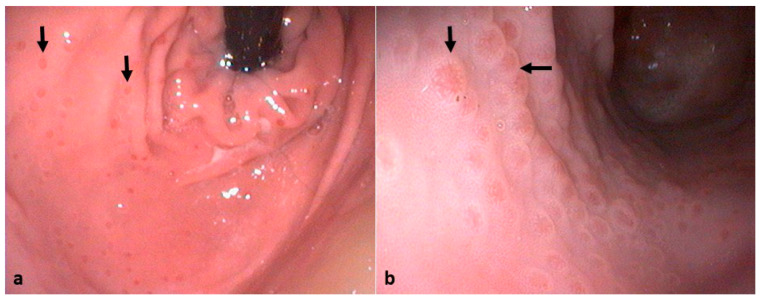
Endoscopic features of gastric mucosa from two dogs with LFH. Note the presence of numerous variably sized gray foci on the surface of the gastric mucosa from the cardia (arrows) (**a**) and body–antrum junctional zones (arrows) (**b**).

Upper digestive endoscopy (UDE) revealed the following findings: lymphoid follicular hyperplasia/nodularity (27/41) (Figure 1), esophageal diverticulum (13/41), esophagitis (9/41), gastric ulcers (9/41), cardiac sphincter dysfunction (6/41), gastric erosions (5/41), and edema of the gastric mucosa (5/41). In some of the cases, changes in the duodenum were also noted such as hypertrophy of the duodenal mucosa (4/41) and duodenitis (3/41). 

### 3.2. Histopathology

**Figure 2:**
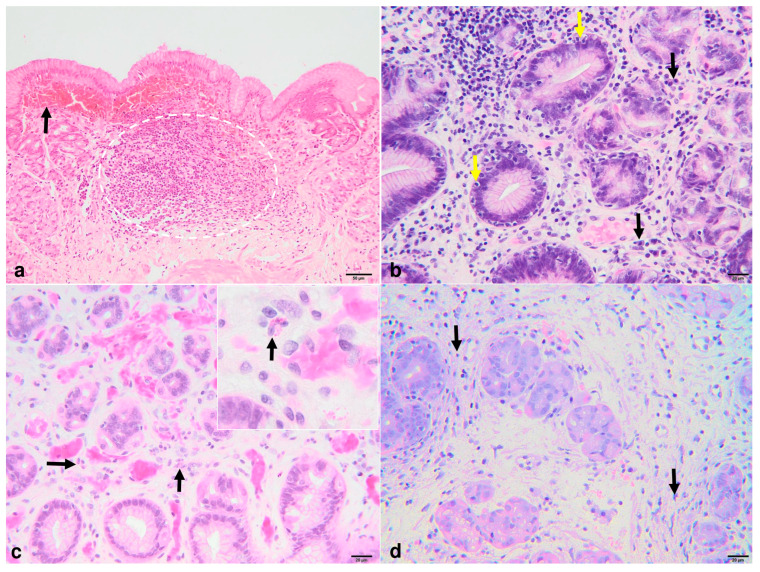
Photomicrographs of GLFH-associated lesions. Notable changes were represented by hemorrhage (black arrow) in the proximity of a small lymphoid follicle (encircled area) (**a**), inflammatory infiltrates represented by lymphocytes both in the interstitial connective tissue as well as intraepithelial (yellow arrow) (**b**), plasma cells (black arrow) (**b**), eosinophils (black arrows and inset) (**c**), and rare neutrophils, and fibrosis (black arrows) (**d**); H&E.

A total of 41 cases with GLFH were assessed, and the distribution of the gastric lesions was the following: 4 in both the antrum and gastric body zones, 13 in the antrum, and 22 in the gastric body (Figure 2a). The number of gastric lymphoid follicles identified in biopsy specimens ranged from 1 to 4. For lymphoid follicles situated in the antrum, the mean diameter was 294.641 μm, with a range between 105.673 μm and 563.280 μm. Regarding the diameter of the follicles located in the gastric body, they exhibited an average diameter of 295.587 μm, ranging from 141.257 μm to 689.52 μm.

Upon assessing the surface area occupied by follicles in the gastric biopsies, following the WSAVA guidelines, it was noted that in both the antrum and gastric body, grade 1 of severity (7 out of 17 in the antrum and 11 out of 26 in the gastric body) was the most prevalent, followed by grade 2 (4 out of 17 in the antrum and 9 out of 26 in the gastric body). Additionally, equal occurrences of grades 0 and 3 were observed in both regions (3 out of 17 in the antrum and 3 out of 26 in the gastric body). 

The associated mucosal lesions found in the antrum included glandular atrophy (10/17 cases), chronic inflammatory infiltrates represented by lymphocyte/plasma cells (16/17 cases) and occasionally eosinophils (Figure 2b,c) and neutrophils, and fibrosis (Figure 2d). Similar inflammatory infiltrates were observed in the gastric body (20/24 cases), but fibrosis (9/24 cases) and glandular atrophy were less severe. Notably, a higher prevalence of cases exhibited surface epithelial injury represented by erosions/ ulcerations. The grading of the lesions found in the antrum and body are outlined in Supplementary File S2. 

The microscopical investigation of the gastric mucosa using H&E, Giemsa, and WS stains revealed the following: 12 cases displayed no evidence of HLOs (−), 20 dogs showed minimal colonization with HLOs (+), 6 cases showed a moderate colonization (++), and 3 dogs presented with a severe degree of HLOs colonization (+++). 

**Figure 3:**
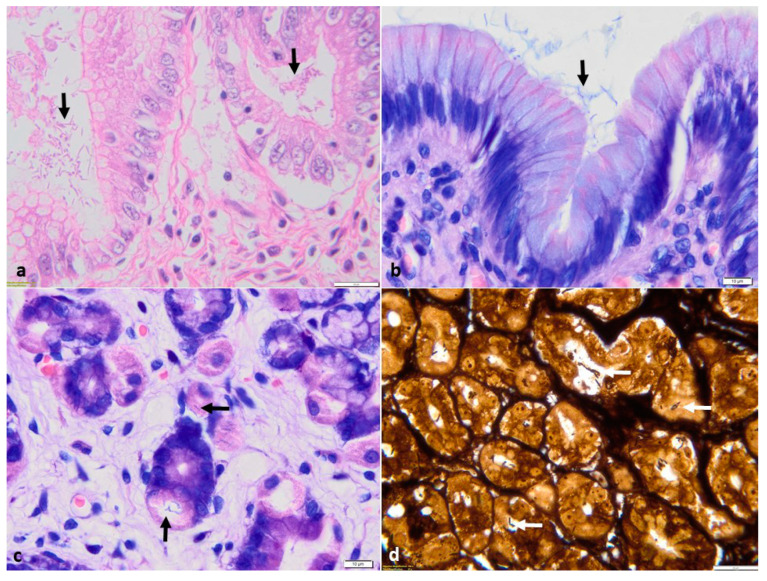
Photomicrographs of *Helicobacter*-like organisms located in the lumen of the gastric glands and foveolae(black arrow) (**a**), mucosal surface (black arrow) (**b**), and cytoplasm of the parietal cells (black and white arrow) (**c**,**d**); H&E stain (**a**), Giemsa stain (**b**,**c**), and WS stain (**d**).

In cases with minimal colonization, spiral bacteria had a multifocal distribution on the gastric mucosa’s surface and within the gastric mucus. Furthermore, in cases of severe infections, HLOs were identified not only on the mucosal surface but also within the gastric crypts and in the cytoplasm of parietal cells (Figure 3). 

### 3.3. Immunohistochemistry

**Figure 4:**
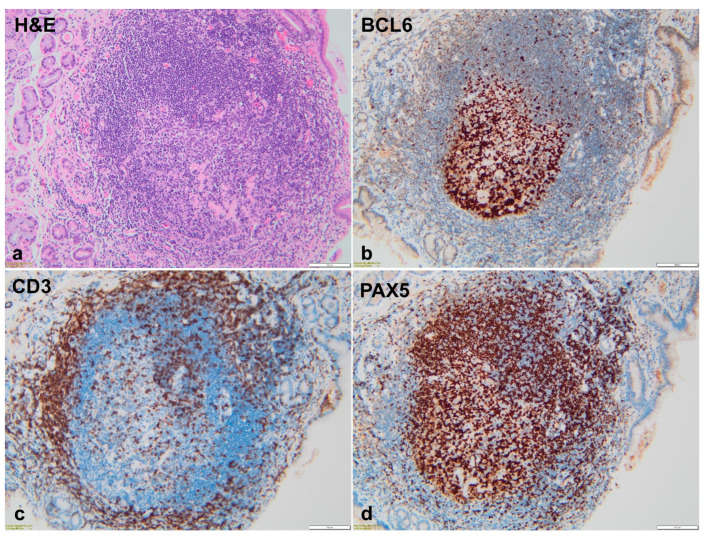
Photomicrographs of a large, hyperplastic, gastric lymphoid follicle (**a**), composed of a light central area (germinative center) and a darker peripheral zone, H&E stain; the peripheral zone is composed of CD3+ T lymphocytes (**c**), and the central area consists of Pax5+ and BCL6+ B lymphocytes (**b**,**d**).

The immunohistochemical evaluation of the gastric lymphoid follicles revealed a distinctive distribution among lymphocyte populations. In the large lymphoid follicles (Figure 4a), the central area (germinal center) exhibited a marked immunolabeling for Bcl6+ and Pax5+ B lymphocytes (Figure 4b,d), displaying their active proliferation. CD3+ T lymphocytes were predominantly situated at the periphery of the lymphoid follicles, with scattered positive cells in the central area (Figure 4c). Conversely, small follicles comprised both CD3+ T lymphocytes and Pax5+ B lymphocytes but lacked the formation of a germinal center with Bcl6+ lymphocytes.

Both the lymphocytic infiltrate within the lamina propria and the intraepithelial lymphocytes were predominantly composed of CD3+ T cells with seldom Pax5 and Bcl6 positive B cells. 

### 3.4. Statistical Analysis

A comparative analysis of the antrum and gastric body regions for the included variables revealed no statistically significant differences between these anatomical regions. Specifically, parameters such as the number of lymphoid follicles, surface epithelial injury, atrophy/fibrosis, and the severity degree of gastric follicle hyperplasia demonstrated no significant variation between the antrum and gastric body (*p* > 0.05). Likewise, the follicle diameter, the severity of HLOs colonization, and lymphoplasmacytic infiltrates within the lamina propria showed no significant differences between these gastric anatomical sites (*p* > 0.05). The median values and interquartile ranges reflected comparable distributions between the two anatomical locations, indicating that the assessed parameters did not exhibit significant anatomical localization-dependent variations (Supplementary File S3).

Spearman’s rank-order correlation was conducted to assess the relationships between the diameters of follicles, inflammation grade, HLOs colonization grade, and the grade of follicular hyperplasia. The results are presented in Table 1.

A positive moderate correlation was found between the diameter of follicles and HLOs (*p* = 0.049) and a positive strong correlation between the diameter of follicles and the grade of follicular hyperplasia (*p* = 0.0001). This suggests that the diameter of follicles increases with HLOs colonization grade and the grade of follicular hyperplasia. 

A multiple linear regression analysis was conducted to examine the effect of the degree of follicular hyperplasia and degree of HLOs colonization on the diameter of the follicles. 

The model yielded an R2 value of 0.423, suggesting that approximately 42.3% of the variance in the follicle diameter is explained by the degree of follicular hyperplasia and the degree of HLOs colonization. This indicates a moderately strong relationship between the grade of follicular hyperplasia and HLOs colonization grade and the diameter of follicles. The significance value (*p* < 0.001) indicates that the effect of follicular hyperplasia on the follicle diameter is highly statistically significant, suggesting that the degree of follicular hyperplasia is a critical factor in determining the diameter of follicles, with a significant and sizable effect. This could have important implications for clinical assessments, indicating that monitoring the degree of follicular hyperplasia could be valuable in predicting follicle size, which may be relevant for diagnostic or treatment considerations.

No statistical significance was observed between the grade of inflammation and the HLOs colonization grade or the grade of follicular hyperplasia.

## 4. Discussion

Lymphofollicular hyperplasia is classified into diffuse (the entire gastrointestinal mucosa) and localized forms, mainly involving the terminal ileum, rectum, or other sites of the gastrointestinal tract [26]. 

The epidemiology and clinical significance of gastric lymphofollicular hyperplasia are poorly characterized in canine patients and its etiology remains unidentified [20]. The goal of this study was to investigate the pathological and immunohistochemical aspects of GLFH in dogs with a clinical history of chronic vomiting and endoscopic nodular gastritis. A correlation between GLFH, associated mucosal changes, and the presence of HLOs was also conducted. 

In our investigation, French Bulldogs were overrepresented (23/41 cases). Affected dogs were mostly males (31/41 cases) and had a mean age of 3.52 years, with younger individuals being particularly susceptible. The data obtained are broadly consistent with a recent study reported by Bienes et al. (2022), in which intact adult male French Bulldogs are more predisposed to develop lymphoid follicle hyperplasia, with males exhibiting follicles of larger diameters than females [19]. These observations are further supported by two other investigations [27,28]. 

The retrospective design of our study limits the understanding of clinical manifestations in affected patients, largely due to incomplete clinical data and the absence of definitive clinical diagnosis. In our study, chronic vomiting remains the most consistent clinical sign reported. It was found that vomiting is typically regarded as a primary clinical sign of GLFH in dogs. However, because GLFH frequently co-occurs with lymphoplasmacytic gastritis, it remains unclear whether GLFH or the latter condition is the primary etiological factor underlying these clinical signs [19]. In the current study, mucosal lymphocytic and plasmocytic infiltrate were found in 87.80% of cases (36/41) with lymphofollicular hyperplasia, but no appreciable significance between the severity of inflammation and grade of follicular hyperplasia nor diameter of the follicles was found.

In our study the most significant endoscopic findings included lymphoid follicular hyperplasia (65.85% of cases), gastric ulcers (21.95%), esophageal diverticulum, and esophagitis. This is in good agreement with recent studies where gastric ulcers accounted for 33% of dogs with GLFH. The authors suggested that ulceration was attributable to *Helicobacter*-like organism infection and the associated lymphocytic immune response [19].

Regarding the evaluation of HLOs colonization by Giemsa and WS stains, 29 out of the 41 cases showed the presence of these organisms in the gastric mucosa, with 20 of these cases classified as mild degree. Furthermore, moderate positive correlation was identified between the follicle diameter and the presence of HLOs. Our findings corroborate those of Bienes et al. (2022), in which a positive correlation between gastric lymphoid follicular hyperplasia (GLFH) and HLOs was observed through histological quantification [19]. However, when the polymerase chain reaction (PCR) was used to evaluate the presence of these bacteria, no such correlation was found [29,30]. This discrepancy may be attributed to the fact that HLOs colonization can be common feature in the stomachs of healthy dogs. The histopathological scoring methods have inherent limitations, including uneven bacterial colonization, bacterial fragmentation due to tissue cross-sections, and the potential for misidentification of other spiral-shaped bacteria, such as *Pseudomonas fluorescens* [31].

Given the early onset of the condition, parallels can be drawn with human pediatric cases, where follicular/nodular antral gastritis is a response to chronic infection with *H. pylori* and other species of *Helicobacter* [23]. Differentiating GLFH from atrophic gastritis, both of which feature lymphoid follicles, is crucial. The key differences lie in the location and size of the follicles within the gastric lamina propria; GLFH is characterized by larger, more superficial follicles, while atrophic gastritis defined by smaller, deeper follicles [32,33]. The inflammatory infiltrate within the lamina propria predominantly consists of lymphocytes and plasma cells. An immunohistochemical analysis revealed a predominance of CD3+ T cells both in the lamina propria and the epithelium [23]. In cheetahs, a similar distribution was observed, with CD4+ T cells located in the lamina propria and CD8α+ T cells in the epithelium. This pattern was more commonly seen in individuals with mild HLOs infections, whereas more severe infections showed a higher prevalence of CD79a+ B cells [34]. 

The gastric mucosa of Beagle dogs experimentally infected with *H. pylori* exhibited infiltration by CD3+ lymphocytes, which were organized into well-structured lymphoid follicles. These follicles contained a germinal center-like area composed of CD21+ lymphocytes, surrounded by a peripheral region predominantly consisting of CD3+/CD4+ T lymphocytes. A few CD8+ T lymphocytes and macrophages were also observed in the cortical areas. In cheetahs, both lymphoid aggregates and follicles contained a central area positive for CD45R+, with scattered T cells present within both the central and peripheral regions [34]. In GLFH of the human gastric mucosa, B lymphoid cells confined to small germinal centers were positive for Bcl-6 but negative for Bcl-2 [35]. As anticipated, in our study, immunohistochemical analysis revealed that the follicles with a germinal center contained a central area of Pax5-positive and Bcl6-positive B lymphocytes, which was surrounded by CD3-positive T lymphocytes. A similar pattern was also observed in a study on gastric mucosa of human patients [24]. 

Ikuse et al. (2018) reported an overexpression of Pax-5 and BCL6 in the gastric mucosa, particularly in the antral region, of *Helicobacter pylori*-infected children [23]. However, the study concluded that Pax-5 and BCL6 did not have a significant impact on the size of the lymphoid follicles. In contrast, CD20 was identified as playing a critical role in the proliferation of memory B cells, which contributes to the enlargement of the germinal center and the development of nodular gastritis. CD20 is a specific marker for the differentiation of pro-B cells into memory B cells, which can be found adjacent to the germinal center. Upon activation, these memory B cells differentiate into plasma cells, thereby promoting the formation and maintenance of secondary germinal centers. These markers have also been implicated in the pathogenesis of gastric cancers. The overexpression of CD20 in the antral mucosa of children with follicular gastritis highlights the need for further research in veterinary medicine to investigate whether analogous changes occur in young dogs.

In contrast to gastric lymphoid follicular hyperplasia (GLFH), gastric MALT lymphoma is a low-grade B-cell lymphoma characterized by diffuse tissue infiltration by small B-cell sheets [35]. Furthermore, in human patients, the histological features of the GLFH were very similar to the background mucosal aspect of the early-stage of MALT-type gastric lymphoma, and *H.pylori* infection may be a high-risk factor in the development of this condition [8]. In dogs, further data collection would be needed to determine the potential role of gastric colonization with HLOs and GLFH in gastric oncogenesis.

## 5. Conclusions

This study provides a comprehensive characterization of gastric lymphofollicular hyperplasia (GLFH) in dogs, affecting predominantly young French Bulldogs. Histopathological analysis revealed a positive correlation between follicle size and both the degree of HLOs colonization and follicular hyperplasia severity, suggesting that HLOs may play an important role in the development of this condition. The investigation of the immunohistochemical pattern of hyperplastic follicles in the canine gastric mucosa may provide the basis for distinguishing this disease from the early stages of gastric follicular lymphoma. Additional studies are required to investigate potential correlations between the development of this condition and specific organisms of the *Helicobacter* genus, as well as to assess its in the progression to neoplastic changes.

## Figures and Tables

**Table 1:** Spearman’s rank-order correlation test.

Variables		Inflammation Grade	HLOs Grade	Grade of Follicular Hyperplasia
Diameter of follicles	Correlation coefficient	0.066	0.345 *	0.662 **
	*p*	0.666	0.049	<0.001
Inflammation grade	Correlation coefficient	-	−0.194	0.021
	*p*	-	0.280	0.892
HLOs grade	Correlation coefficient	−0.194	-	0.276
	*p*	0.280	-	0.120

* Correlation is significant at the 0.05 level (2-tailed). ** Correlation is significant at the 0.01 level (2-tailed).

## Data Availability

The original contributions presented in this study are included in the article/Supplementary Material. Further inquiries can be directed to the corresponding author.

## Supplementary Materials

The following supporting information can be downloaded at https://www.mdpi.com/article/10.3390/ani14243605/s1, Table S1. Quantification of the lesion in the gastric antrum. Table S2. Quatification of the lesion in the gastric body.
