# Supplementary material for: Pathological and Immunohistochemical Characterization of Follicular Gastritis (Gastric Lymphofollicular Hyperplasia) in 41 Dogs
Source: Animals (Basel). 2024 Dec 14;14(24):3605. doi: 10.3390/ani14243605 (PMC11672492; doi:10.3390/ani14243605)
Supplement: Supplementary file 1 [file animals-14-03605-s001.zip › Supplementary file 1.pdf]

	Identification number	Vomiting (-/+)	Diarrhea (-/+)	Anorexia (-/+)	Weight loss (-/+)	Other clinical signs	Endoscopic changes
1	26850	-	-	-	-	-	gastric erosions, esophageal diverticulum, LFH
2	26876	-	-	-	-	-	gastric erosions, duodenitis
3	32603	-	-	-	-	-	gastric erosions, duodenitis
4	35344	-	-	-	-	-	esophageal diverticulum, esophagitis, cardia sphincter dysfunction, LFH
5	34746	-	-	-	-	-	esophageal diverticulum, esophagitis, cardia sphincter dysfunction, LFH
6	34775	-	-	-	-	-	esophageal mucosal edema, cardia sphincter dysfunction
7	94209	+	-	-	-	regurgitation	esophageal diverticulum, esophagitis and edema of the gastric mucosa, LFH
8	94333	-	-	-	-	-	esophageal diverticulum, gastric mucosal hyperplasia, gastric ulcers, LFH
9	94629	-	-	-	-	-	LFH
10	95005	-	-	-	-	-	esophageal diverticulum, hiatal hernia, hypertrophy and edema of the duodenal mucosa, LFH

11	95038	+	+	-	-	-	gastric ulcers, LFH
12	95180	-	-	-	-	-	NL
13	95495	-	-	-	-	-	esophageal diverticulum, hypertrophy of the duodenal mucosa, LFH
14	95555	-	-	-	-	-	esophageal diverticulum, esophagitis, hiatal hernia, gastric mucosal edema, LFH
15	95591	-	-	-	-	-	-
16	95657	-	-	-	-	-	esophageal diverticulum, esophagitis, LFH, gastric ulcers
17	95702	-	-	-	-	-	foreign body
18	95764	-	-	-	-	-	esophageal diverticulum, esophagitis and dysfunction cardia sphincter, LFH
19	95781	-	-	-	-	-	gastric dilatation, nodular esophagitis, LFH
20	95822	-	-	-	-	-	LFH
21	95852	-	-	-	-	-	esophageal diverticulum, edema and hemorrhage of the gastric mucosa, LFH
22	95856	-	-	-	-	-	esophageal diverticulum, esophagitis, hyperplasia of the

							duodenal mucosa
23	95816	-	-	-	-	-	LFH
24	95883	-	-	-	-	-	gastroesophageal reflux, esophagitis, esophageal diverticulum, cardia sphincter dysfunction, LFH
25	95908	-	-	-	-	tonsillitis	gastric ulcers, LFH
26	95998	-	-	-	-	-	pyloric obstruction , gastric erosions, hypertrophy of the duodenal mucosa, LFH
27	96087	-	-	-	-	-	NL
28	96290	-	-	-	-	tonsillitis	gastric ulcers
29	96305	-	-	-	-	-	cerebriform aspect of the mucosa, LFH
30	96440	-	-	-	-	-	NL
31	96459	-	-	-	-	-	cardia sphincter dysfunction, gastric ulcers, LFH
32	96544	-	-	-	-	-	erosions of the gastric mucosa, duodenitis, LFH
33	96587	+	-	-	-	-	gastric ulcers

34	96805	+	-	-	-	-	Edema, congestion and keratinization of the gastric mucosa, LFH
35	6395	-	-	-	-	-	NL
36	6666	+	-	-	-	-	hiatal hernia, LFH
37	6730		-	-	-	-	chronic gastritis
38	6752	+	-	-	-	-	chronic gastritis, LFH
39	7516	+	-	-	-	-	gastric mucosal edema
40	7587	-	-	-	-	-	LFH
41	7830	+	-	-	-	-	gastric ulcers, LFH

*LFH -lymphoid follicular hyperplasia
NL - no lesions
