# Supplementary material for: Pathological and Immunohistochemical Characterization of Follicular Gastritis (Gastric Lymphofollicular Hyperplasia) in 41 Dogs
Source: Animals (Basel). 2024 Dec 14;14(24):3605. doi: 10.3390/ani14243605 (PMC11672492; doi:10.3390/ani14243605)
Supplement: Supplementary file 1 [file animals-14-03605-s001.zip › Supplementary file 2.pdf]

Table 1. Quantification of the lesion in the gastric antrum

		Morphological features (GRADE)			Inflammation (GRADE) (antru)					
	Identification number	Surface epithelial injury	Epithelial hyperplasia	Fibrosis/gland atrophy	IEL	LP, L+P	LP, Eos	LP, Neut	LP, Other cells	GFH
1	**26850**	0	0	3	1	2	1	0	0	1
2	**32603**	2	2	1	1	1	0	0	0	3
3	**94333**	1	1	0	0	1	1	0	0	0
4	**95495**	0	0	1	0	1	0	0	0	1
5	**95657**	0	0	0	0	0	0	0	0	0
6	**95764**	0	0	0	0	1	0	0	0	1
7	**95852**	0	0	2	0	1	0	0	0	1
8	**95856**	0	0	1	1	1	0	0	0	3
9	**95883**	0	0	2	1	2	0	0	0	2
10	**95998**	0	0	1	1	2	0	0	0	3
11	**96290**	0	0	0	0	1	0	0	0	2
12	**96305**	0	0	0	0	1	0	0	0	2
13	**96440**	0	0	0	0	1	0	0	0	1
14	**96544**	0	0	0	0	1	0	0	0	1
15	**6395**	0	0	1	0	1	0	0	0	0
16	**6666**	1	0	2	1	1	1	0	0	2
17	**6730**	0	0	1	0	1	0	0	0	1

*IEL - intraepithelial lymphocytes
LP - laminapropria
Eos - eosinophilic
Neut - neutrophilic
GFH - grade of follicular hyperplasia

Table 2. Quatification of the lesion in the gastric body

		Morphological features (GRADE)			Inflammation (GRADE)					
	Identification number	Surface epithelial injury	Gastric pit epithelial injury	Fibrosis/gland nest/atrophy	IEL	LP, L+P	LP,Eos	LP, Neut	LP, Other cells	GFH
1	**35344**	0	0	0	0	1	1	0	0	0
2	**26876**	0	0	0	1	1	0	0	0	2
3	**32603**	0	0	0	0	1	0	0	0	3
4	**34746**	0	0	0	1	0	0	0	0	0
5	**34775**	0	0	1	1	1	0	0	0	1
6	**94209**	0	0	0	1	0	0	0	0	1
7	**94629**	0	0	1	0	1	1	0	0	1
8	**95005**	0	0	0	0	0	0	0	0	3
9	**95038**	0	0	1	1	1	0	0	0	2
10	**95180**	0	0	0	0	1	0	0	0	1
11	**95555**	0	0	1	1	1	0	0	0	3
12	**95591**	2	1	1	1	1	0	0	0	1
13	**95702**	1	0	0	0	0	0	0	1	2
14	**95781**	0	1	0	1	1	0	0	0	1
15	**95822**	2	2	1	0	1	0	0	0	2
16	**95816**	1	0	0	0	1	0	0	0	1
17	**95883**	0	0	2	1	2	0	0	0	0
18	**95908**	1	1	1	1	1	0	0	0	1
19	**96087**	0	1	1	1	1	0	0	0	2
20	**96290**	0	0	0	0	1	0	0	0	2
21	**96459**	2	1	0	0	1	0	1	0	2
22	**96587**	0	0	0	0	2	0	0	0	1
23	**96805**	0	0	0	2	2	0	0	0	1
24	**6395**	0	0	0	0	1	0	0	0	0
25	**6752**	0	1	2	1	1	0	0	0	2

26	**7516**	0	0	1	2	1	0	0	0	2
27	**7587**	2	1	1	1	1	0	0	0	2
28	**7830**	0	0	0	0	1	0	1	0	1

*IEL - intraepithelial lymphocytes
LP - lamina propria
Eos - eosinophilic
Neut - neutrophilic
GLFH - grade of follicular hyperplasia
