# Supplementary material for: Pathological and Immunohistochemical Characterization of Follicular Gastritis (Gastric Lymphofollicular Hyperplasia) in 41 Dogs
Source: Animals (Basel). 2024 Dec 14;14(24):3605. doi: 10.3390/ani14243605 (PMC11672492; doi:10.3390/ani14243605)
Supplement: Supplementary file 1 [file animals-14-03605-s001.zip › Supplementary file 3.pdf]

**Variable**	**Antrum Median (IQR)**	**Body Median (IQR)**	**Wilcoxon Statistic**	**Wilcoxon p-value**	**Kruskal-Wallis Statistic**	**Kruskal-Wallis p-value**
**No GF**	0.0 (0.0 - 1.0)	1.0 (0.0 - 2.0)	313.0	0.182	N/A	N/A
**Surface Epithelial Injury**	0.0 (0.0 - 0.5)	0.0 (0.0 - 0.0)	0.0	0.317	N/A	N/A
***Helicobacter* Grade**	2.0 (1.75 - 2.25)	2.0 (1.75 - 2.25)	N/A	N/A	0.0	1.0
**Fibrosis/Gland Atrophy**	1.0 (0.75 - 1.25)	0.0 (0.0 - 0.5)	0.0	0.157	N/A	N/A
**Lymphoplasmacytic Inflammation**	1.0 (1.0 - 1.25)	1.0 (1.0 - 1.25)	N/A	N/A	0.0	1.0
**GFH**	2.0 (1.5 - 2.25)	1.0 (0.0 - 2.25)	0.0	0.317	N/A	N/A
**Diameter GF**	347.539 (261.787 - 431.365)	317.957 (188.242 - 451.323)	4.0	0.875	N/A	N/A

* GF- gastric follicles

GFH- grade of follicular hyperplasia
